# Clones of cells switch from reduction to enhancement of size variability in *Arabidopsis* sepals

**DOI:** 10.1242/dev.153999

**Published:** 2017-12-01

**Authors:** Satoru Tsugawa, Nathan Hervieux, Daniel Kierzkowski, Anne-Lise Routier-Kierzkowska, Aleksandra Sapala, Olivier Hamant, Richard S. Smith, Adrienne H. K. Roeder, Arezki Boudaoud, Chun-Biu Li

**Affiliations:** 1 Theoretical Biology Laboratory, RIKEN, Wako 351-0198, Japan; 2 Laboratoire de Reproduction et Développement des Plantes, Universiteé de Lyon, ENS de Lyon, Université Claude Bernard Lyon 1, INRA, CNRS, 46 Allée d'Italie, 69364 Lyon Cedex 07, France; 3 Department of Comparative Development and Genetics, Max Planck Institute for Plant Breeding Research, Carl-von-Linné-Weg 10, 50829 Köln, Germany; 4 Weill Institute for Cell and Molecular Biology and Section of Plant Biology, School of Integrative Plant Sciences, Cornell University, Ithaca, NY 14853, USA; 5 Department of Mathematics, Stockholm University, 106 91 Stockholm, Sweden

**Keywords:** Cell size variability, Clone, Size uniformization, Cell growth heterogeneity

## Abstract

Organs form with remarkably consistent sizes and shapes during development, whereas a high variability in growth is observed at the cell level. Given this contrast, it is unclear how such consistency in organ scale can emerge from cellular behavior. Here, we examine an intermediate scale, the growth of clones of cells in *Arabidopsis* sepals. Each clone consists of the progeny of a single progenitor cell. At early stages, we find that clones derived from a small progenitor cell grow faster than those derived from a large progenitor cell. This results in a reduction in clone size variability, a phenomenon we refer to as size uniformization. By contrast, at later stages of clone growth, clones change their growth pattern to enhance size variability, when clones derived from larger progenitor cells grow faster than those derived from smaller progenitor cells. Finally, we find that, at early stages, fast growing clones exhibit greater cell growth heterogeneity. Thus, cellular variability in growth might contribute to a decrease in the variability of clones throughout the sepal.

## Abstract (precis)

**Summary:** Growth analyses of *Arabidopsis* sepals identify a tipping point in organ development, at which clones of cells change their growth pattern from size uniformization to size variability enhancement.

## INTRODUCTION

As in most living multicellular organisms, plant organs are reproducible; organs have their own characteristic sizes and shapes, making them landmarks for species identification in botany. Naively, we might expect reproducible organs to arise from uniform cells with constant sizes and shapes like tiles in a floor. However, live imaging demonstrates that in many cases, cell sizes, growth rates and directions exhibit considerable variability (Roeder et al., 2010, 2012; Hong et al., 2016; Elsner et al., 2012; Kierzkowski et al., 2012; Tauriello et al., 2015; Uyttewaal et al., 2012). For instance, the timing and geometry of cell division is variable in organs with stereotypical shapes (Roeder et al., 2010; Besson and Dumais, 2011). These results raise the question of the contributions of such cellular noise and variability to organ size/shape consistency (Meyer and Roeder, 2014; Hong et al., 2016).

The complexity of the relationship between cells and organs can be illustrated with a few famous and mysterious biological examples. The first is the ‘regulative egg’ example; in specific early stages, when half of the early *Xenopus* embryo is removed, the remaining half produces a complete tadpole of half size (Spemann and Mangold, 1924; Cooke, 1975). This suggests that cell fate can be determined by the relative location within the embryo. In that scenario, cells would not be fully autonomous but instead subordinate to the whole shape and function of the embryo. A second example is ‘compensation’; when a mutation inhibits cell division and consequently reduces the number of cells in the organ, and individual cells compensate that loss by increasing their size to produce an organ of nearly the correct size and shape (Tsukaya, 2003). This phenomenon of compensation suggests that organs have a global size/shape-sensing mechanism, which makes cell growth subordinate to the whole organ size/shape. Yet, as mentioned above, cells retain an ability to display variable growth rates, which suggests that cells are also autonomous to a large extent (Asl et al., 2011; Elsner et al., 2012). Therefore, we are left with a picture in which development results from a balance between the organismal theory (Kaplan and Hagemann, 1991; cell behavior is the consequence of the organ behavior) and the cell theory (organ behavior is the consequence of cell behavior). To shed light on the mechanisms balancing individual and collective behaviors in cell growth, we chose to focus on an intermediate scale, groups of cells, using a kinematic approach.

Here, we focus on a clone (i.e. a group of related cells that descend from a single progenitor cell) in *Arabidopsis* sepals as an attempt to identify a unifying mechanism, which could also be compatible with both the cell theory and the organismal theory. Interestingly, Tauriello et al. (2015) used a kinematic approach to extract the growth of the clones in order to determine general properties of the growth curves. Surprisingly, they found that the sizes of different clones follow the same sigmoidal function of time, albeit with a stochastic timing of maximal growth rate, implying that the clones do not grow ‘freely’ but are instead constrained. Because these growth curves start from different initial cell sizes, the exact contribution of initial size distribution in such growth patterns becomes a central question. In this study, we investigated the detailed kinematics and relationships between the growth behaviors and starting sizes of clones in *Arabidopsis* sepals.

## RESULTS

### Clones switch growth patterns from size uniformization to size variability enhancement

**Fig. 1.**
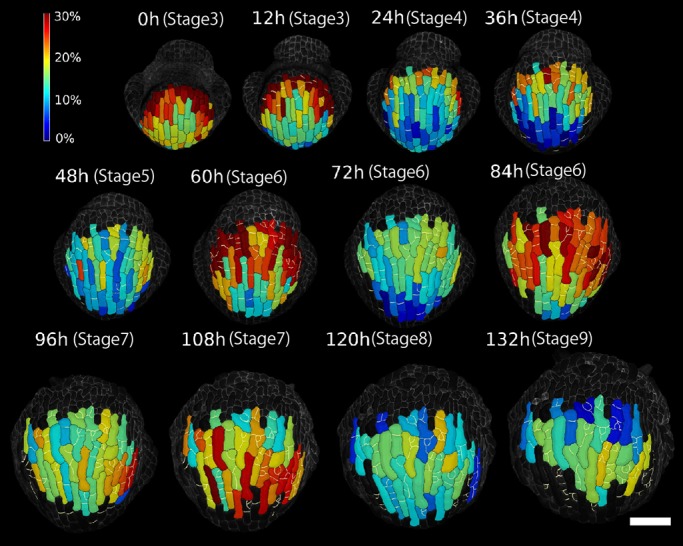
**Spatio-temporal growth pattern of clones.** Heat map of areal growth of clones (*A*_*t*+Δ*t*_−*A*_*t*_)/*A*_*t*_×100 (%) over consecutive 12 h intervals for flower wt-a1. The clones are outlined in black. New cell walls resulting from divisions within the clone are marked in white. Note that around flower stage 5 to 7, there is a higher growth rate at night and a relatively lower growth rate during the day. Scale bar: 50 μm.

First, we investigated the relationship between the initial sizes of the clones and their growth rates in developing *Arabidopsis* sepals. Here, a clone refers to the progenitor cell and all of its descendants, and hereafter we use ‘an initially small (or large) clone’ for a clone descended from a small (or large) progenitor cell. We tested whether the sizes of the clones within the sepal become more uniform (size uniformization) or more variable (size variability enhancement) over time. Live imaging data from two laboratories (five wild-type sepals), previously reported in Hervieux et al. (2016), were considered. In this study, cells were outlined with plasma membrane markers and the entire sepal was imaged every 12 h or 24 h. We considered the growth of the entire clone as a unit, and ignored divisions of cells within the clone. The growth of individual cells will be discussed in the section headed ‘Individual cell growth heterogeneity is positively correlated with the growth of clones at each time step’. To extract the outline and follow the growth of clones, we used analysis and visualization software, MorphoGraphX (MGX) (Barbier de Reuille et al., 2015; see Materials and Methods), for cell segmentation, lineage tracking and area calculations. We defined the clone area at time *t* as *A*_*t*_, and calculated the relative areal growth of the clone as (*A*_*t*+Δ*t*_−*A*_*t*_)/*A*_*t*_×100 (%) (Fig. 1). The growth patterns in all analyzed sepals were qualitatively similar (Fig. S1A-D).

**Fig. 2.**
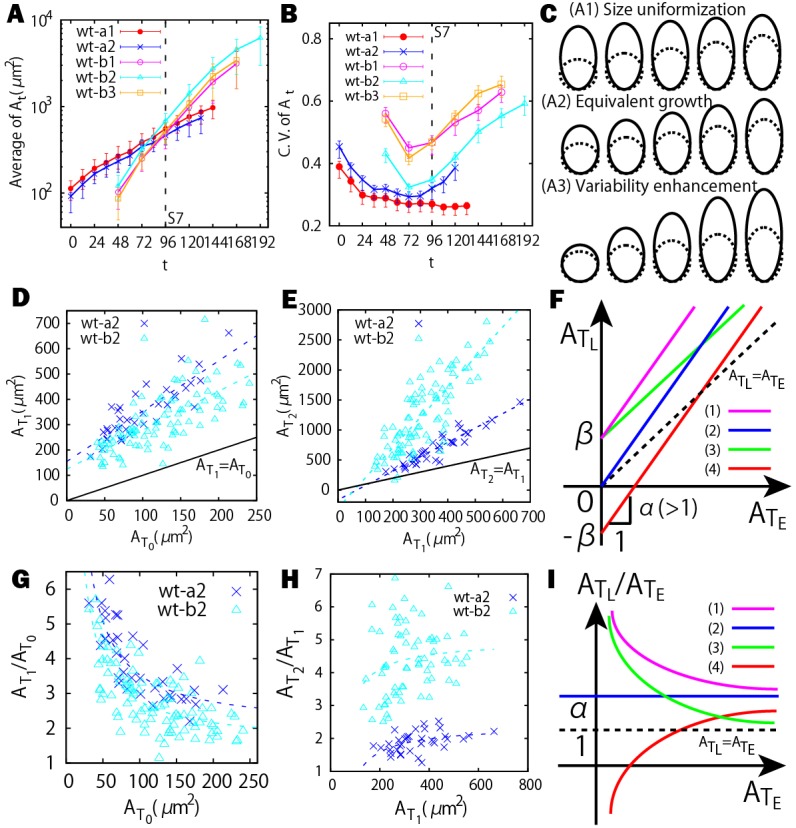
**Size uniformization and variability enhancement in clones.** (A) The average area of the clones over time. (B) CV to quantify area variability. Error bars represent the 50% confidence interval (see Materials and Methods). The CV decreases first and subsequently increases. Note that the sepal wt-a1 does not show an obvious increase, which could be due to the fact that initial cells in wt-a1 were imaged mainly from the tip to the middle part of the sepal. (C) Schematic illustration of the three growth trends with earlier clone size (dashed lines) and later clone size (solid lines): size unifomization (A1), initially smaller clones grow faster; equivalent growth (A2), clones grow independent of their initial sizes; variability enhancement (A3), initially larger clones grow faster. (D,E) 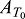 versus 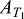 plot for the clones (D), and 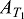 versus 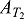 plot (E). The dashed lines are linear fittings of the data points in the plot of 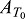 versus 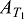. (F) Examples of different growth trends showing linear correlation between the area at a later time 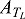 and those at an earlier time 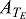. Curves 1 (green) and 3 (pink) illustrate uniformization, curve 2 (blue) illustrates equivalent growth, and curve 4 (red) illustrates variability enhancement. (G,H) 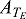 versus 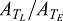 plot for the clones at *T*_0_ versus those at *T*_1_ (G), and at *T*_1_ versus those at *T*_2_ (H). The dashed lines in G and H are fittings of the data points corresponding to those of D and E, respectively. (I) Examples of different growth trends between 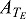 and 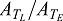. The line colors are the same as in F. Size uniformization (variability enhancement) corresponds to the negatively (positively) correlated case.

Our live imaging of the sepals initiates shortly after the sepal primordia have formed on the flanks of the floral meristem, when the cells are relatively small in size and have not yet differentiated into specialized epidermal cell types. As reported in our previous study (Hervieux et al., 2016), the sepal first undergoes fast growth at the tip (stage 3-4 in Fig. 1). The maximal growth then gradually moves down to the middle (stage 5-7) and bottom of the sepal (stage 8-9) in Fig. 1 (see also Fig. S1A-D). The general growth trend in area of the clones in each sepal can be captured by the average of *A*_*t*_ (Fig. 2A). Although sepals from different laboratories (wt-a1, wt-a2, wt-b1, wt-b2 and wt-b3 in Fig. 2A) are slightly different because of different plant culture conditions, sepals within a given laboratory display comparable growth curves. In Fig. 2 and Table 1, we provide the flower stages of the sepals determined based on Smyth et al. (1990) (see also Materials and Methods).

**Table 1.**
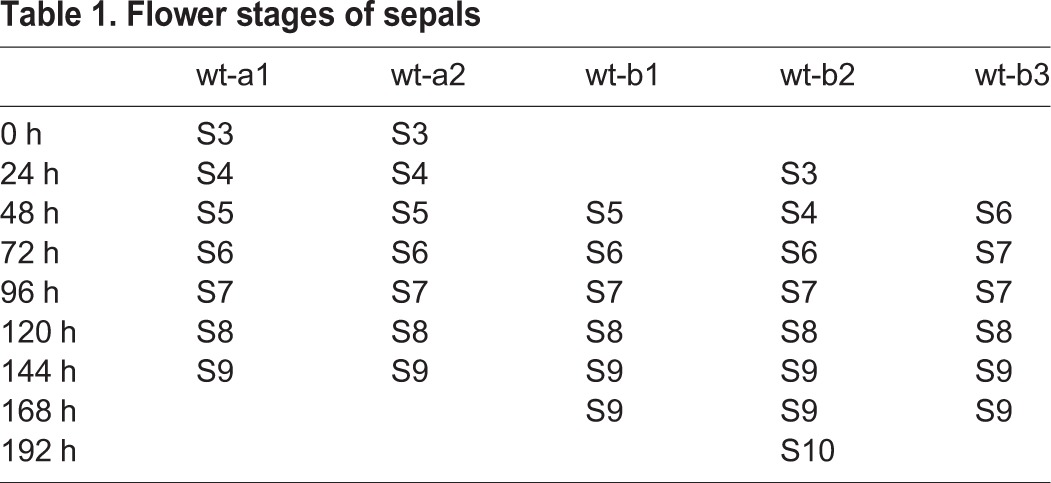
**Flower stages of sepals**

Next, we analyzed how the variability in clone size changes over the development of the sepal. To do so, we calculated the coefficient of variation (CV, defined as standard deviation divided by mean) of *A*_*t*_ as a function of *t* (Fig. 2B). There are three possible trends in the area variability of the clones quantified by the CV (Fig. 2C): (A1) size uniformization, during which initially smaller clones grow faster to catch up with the larger clones (upper panel), leading to a decrease of CV; (A2) equivalent growth, during which the clones grow homogeneously and independently of their initial sizes (middle panel), leading to an unchanged of CV; (A3) variability enhancement, during which initially larger clones grow faster to outrun the smaller clones, leading to an increase in the CV (lower panel). We found that, initially, the CV for each flower decreases, indicating that the clones undergo size uniformization. Then, the growth of clones undergoes a stereotypical transition, after which the CV increases, indicating that the clones switch to size variability enhancement. Thus, we found that there is a tipping point at which the clones make the transition from size uniformization to size variability enhancement during sepal development.

To verify the switch from size uniformization to variability enhancement, we next determined how the area of each clone measured at an early time relates to its area at a later time. We performed this comparison during both the size uniformization stage and the variability enhancement stage. To do so, we first defined some relevant times, *T*_0_, *T*_1_ and *T*_2_, in our analysis. *T*_0_ denotes an early time in our live imaging between stage 3 and 6 (Tables 1 and 2). *T*_1_ denotes the time when the CV reaches its local minimum, i.e. the tipping point. *T*_2_ denotes a later time point after *T*_1_ that is typically around flower stage 8 (Tables 1 and 2). We can measure the cumulative growth ratio by dividing the final clone size by the initial clone size. For size uniformization, we expected that the initially small clones would have higher cumulative growth ratios than the initially large clones [i.e. a negative correlation between 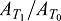 and 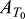, as depicted in the hypothetical graph Fig. 2I (curves 1 and 3)]. As expected, a negative correlation between 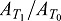 and 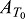 was observed in the real data from [*T*_0_, *T*_1_], verifying that size uniformization occurs before the tipping point (Fig. 2G). By contrast, for size variability enhancement, we expected that initially small clones have lower cumulative growth ratios than the initially large clones [i.e. a positive correlation between 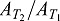 and 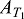, as depicted in the hypothetical graph Fig. 2I (curve 4)]. This positive correlation between 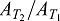 and 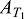
was observed after the tipping point *T*_1_ in the real data, verifying that size variability enhancement occurs.

**Table 2.**
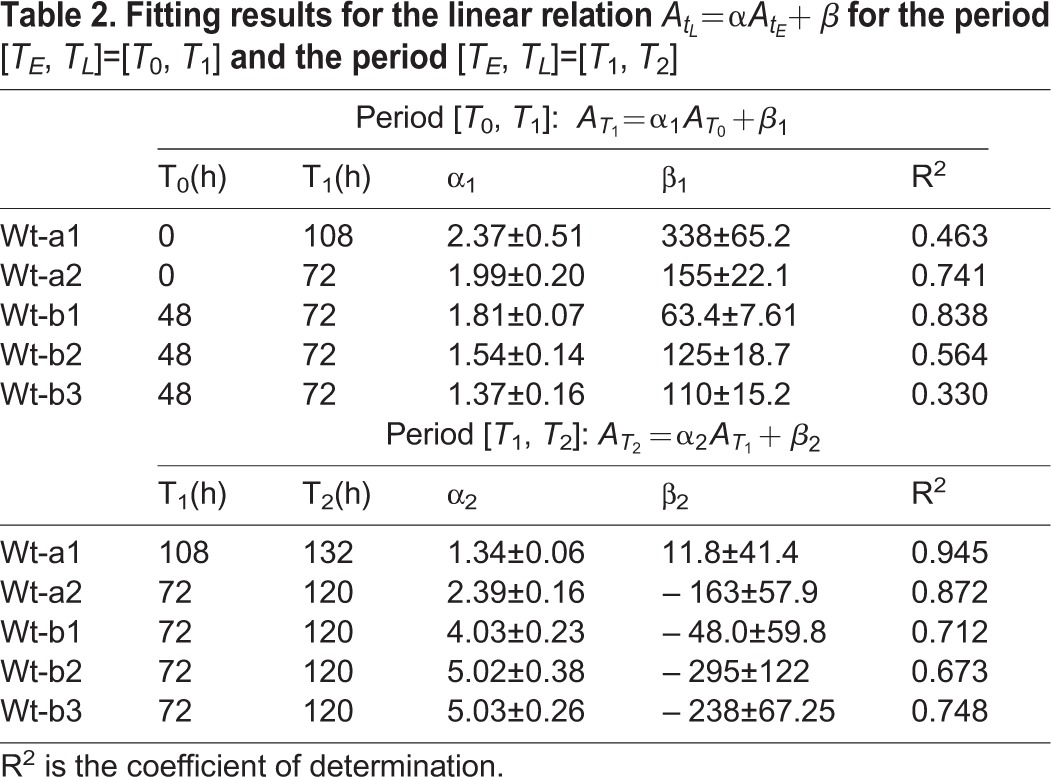
**Fitting results for the linear relation** 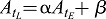 **for the period** [*T*_*E*_, *T*_*L*_]=[*T*_0_, *T*_1_] **and the period** [*T*_*E*_, *T*_*L*_]=[*T*_1_, *T*_2_]

We next further explored the mathematical relationship between the area of each clone measured at an early time and those at a later time in order to find the key parameter distinguishing the size uniformization from the size variability enhancement growth modes. During the size uniformization (A1) period, [*T*_0_, *T*_1_], we observe a linear correlation between the clone area at *T*_0_ (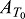) and the clone area at *T*_1_ (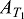) (Fig. 2D). We observe a different linear correlation between clone area at *T*_1_ (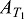) and the clone area at *T*_2_ (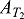) during the variability enhancement (A3) period [*T*_1_, *T*_2_] (Fig. 2E). Moreover, a phenomenological model, which verified that the underlying sigmoidal growth curves of these clones give rise to these empirically observed linear relationships, is provided in the Materials and Methods (see also Fig. S2). Because we observed different linear correlations in 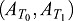 and 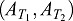 (Fig. 2D,E), we expected that the key parameter for the transition between size uniformization and variability enhancement could be embedded in the linear equation relating clone area at the earlier time (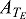) to clone area at the later time (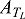), i.e. 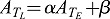 (Fig. 2F). All clones increase their areas from *T*_*E*_ to *T*_*L*_; therefore, the parameter α is greater than or equal to 1. In this framework, the cumulative growth ratio (i.e. final clone size divided by initial clone size), defined as 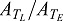, can be easily derived as 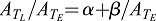. As shown in the hypothetical graphs (Fig. 2F,I), the size uniformization case (A1) occurs when *β*>0 (e.g. curves 1 and 3). The equivalent growth case (A2) (clone growth does not depend on clone size, i.e. 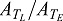 is independent of 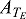) corresponds to *β*=0 (curve 2). The variability enhancement case (A3) (initially larger clones grow faster i.e. 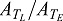 positively correlates with 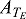) occurs when *β*<0 (e.g. curve 4).

The fitted parameters before the tipping point (*α*_1_, *β*_1_) satisfy *α*_1_>0 and *β*_1_>0 for uniformization (Table 2). On the other hand, after the tipping point (*α*_2_, *β*_2_) satisfy *α*_2_>0 and *β*_2_<0 for the sepals wt-a2, wt-b1, wt-b2 and wt-b3, consistent with variability enhancement. However, *β*_2_>0 for the sepal wt-a1 because wt-a1 shows more or less A1 or A2 (Fig. S3D), consistent with the CV analysis showing that wt-a1 remains in uniformization and does not reach the tipping point (Fig. 2B). To summarize, we concluded that, during sepal development, a size uniformization mechanism reduces the clone area variability at early times before a switch to a different behavior at a tipping point, after which variability enhancement results in subsequent increase in clone area variability. In addition, we find that the parameter *β* corresponding to the *y*-intercept in the plot of 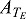 versus 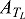 (Fig. 2F) provides an effective parameter for identifying such a growth transition.

### Size uniformization occurs everywhere in the sepal

**Fig. 3.**
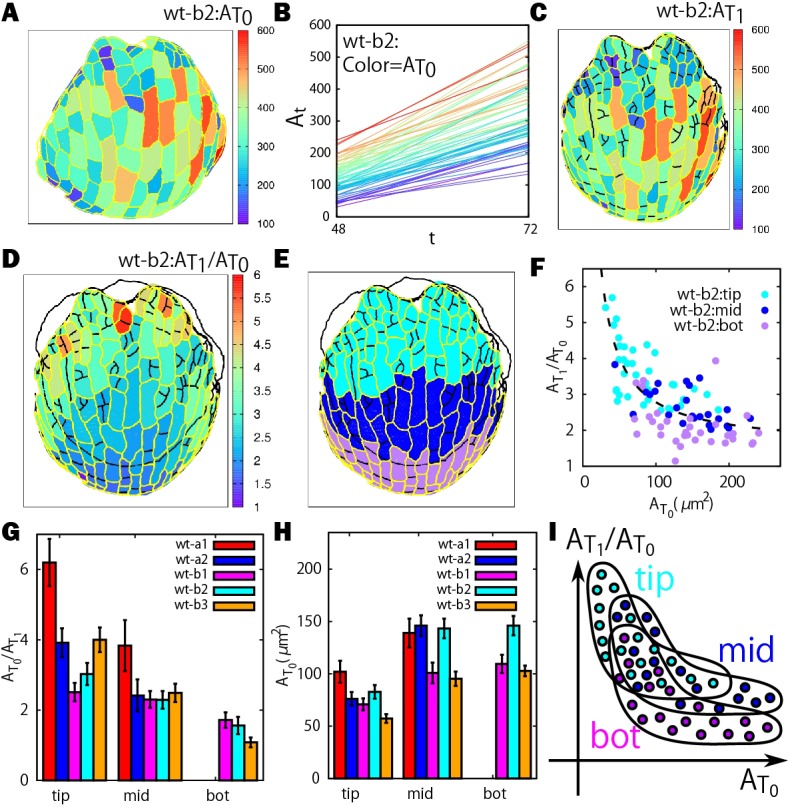
**Size uniformization occurs everywhere in the sepal.** (A) Heat map of the initial cell area 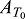. (B) Growth curves of clones colored according to their initial size 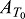. (C) Heat map of the size of the clone 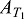. (D) Heat map of the cumulative growth ratio 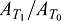, showing a continuous spatial trend from tip to bottom. (E) Classification of tip (cyan), middle (blue) and bottom (purple) regions. (F) Plot of cumulative growth ratios 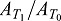 versus initial area 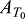 for the clones in each region. (G,H) Histograms of cumulative growth ratios (G) and initial area (H) for the three regions. (I) Schematic illustration of positional dependence of size and growth.

One of the implications from the linear positive correlation between earlier clone sizes and the later ones in both the size uniformization and variability enhancement (Fig. 2D,E) is that the rank of the clone sizes is largely preserved during sepal development. This means that although smaller clones grow faster in the size uniformization stages, their growth rates are not fast enough to catch up with, or even outrun, the initially larger clones. We wondered whether positional information played a role in the ordering of the clone size. To test this, we tracked the areas of the clones *A*_*t*_ in both space and time during the size uniformization stages (Fig. 3A,B) by coloring the growth curves in *A*_*t*_ according to their initial size at time *T*_0_ (Fig. 3B). The color gradient at earlier times is largely preserved at later times (Fig. 3B; Fig. S4), confirming again that the size order of the clones is largely preserved. At the initial time *T*_0_, smaller cells are distributed toward the top part of the sepal and the larger ones toward the bottom. This is because the cells in the top part are farther along in their development and have started dividing at the early flower stages. As expected, the spatial distribution of size is not strongly affected by growth, and the cells at the tip remain smaller than those at the bottom of the sepal at a later time *T*_1_ (Fig. 3C; Fig. S4).

Next, we looked at the spatial distribution of the cumulative growth ratio 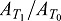 on the sepal (Fig. 3D). There is a continuous change from the high values in 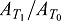 at the tip part to the lower values at the bottom part for all sepals analyzed. To investigate whether the size uniformization occurs only in specific regions (e.g. tip) of the sepal, we manually divided the sepal into the tip, middle and bottom regions as indicated by cyan, blue and purple colors, respectively (Fig. 3E; Fig. S5). 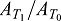 negatively correlated with 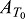 for clones from each region, showing that size uniformization occurs throughout the sepal (Fig. 3F; Fig. S5). The plots from the different regions were slightly shifted relative to one another, as expected, given the overall trends of growth in the sepal; namely, tip clones have smaller 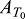 but larger 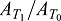, whereas bottom clones have larger 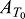 but smaller 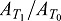 (see also Fig. 3G,H). Although the distributions of 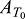 and 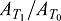 are slightly different, as schematically illustrated in Fig. 3I, our results indicate that size uniformization occurs as a global mechanism in the developing sepal and positional information does not play a significant role in regulating the size of the clones during the size uniformization stages. During the variability enhancement stages, we could not find a characteristic trend (Fig. S6).

### The initial size of clones correlates with the growth of clones at each time step

**Fig. 4.**
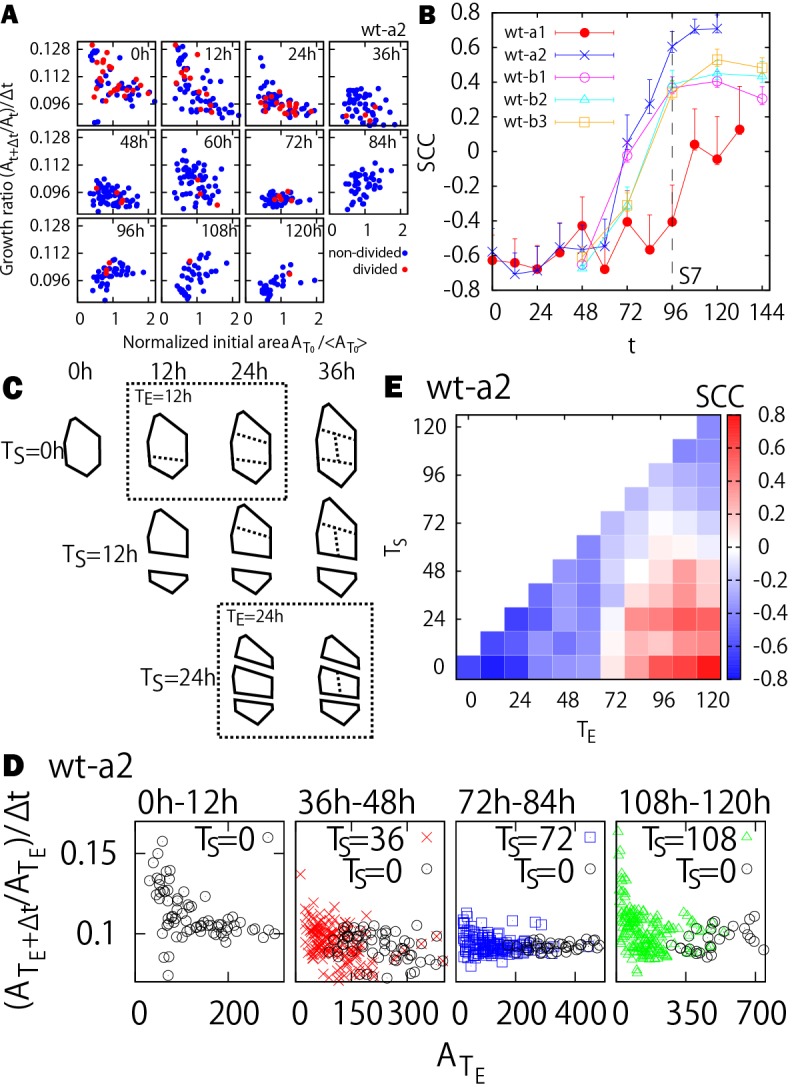
**Size uniformization depends on the starting time of the clone.** (A) Plot of growth ratio at each time step (*A*_*t*+Δ*t*_/*A*_*t*_)/Δ*t* versus normalized initial area 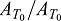 for the sepal wt-a2. Here, the normalized initial area is considered for a better visual comparison of areas at different time points. The dividing and nondividing clones are colored in red and blue, respectively. (B) SCCs for the plots in A. The SCCs change from negative to positive at around stage 6-7 (72-96 h). (C) Schematic illustration of the definition of the clone from starting time T_S_. The dashed outline boxes show some examples of the definition (*T*_*E*_=12 h for *T*_*S*_=0 or *T*_*E*_=24 h for *T*_*S*_=24). (D) Size and growth rates of the different clones. (E) SCCs for different combination of *T*_*E*_ and *T*_*S*_.

As the size uniformization does not depend on the location of clones, we then asked whether there is gradual or sudden temporal change in the correlation between the initial clone size 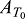 and the growth ratio (*A*_*t*+Δ*t*_/*A*_*t*_)/Δ*t*. In other words, we examined the correlation in short time intervals, in which Δ*t* is 12 h or 24 h (compared with the longer time intervals analyzed in the previous sections). The initial sizes of clones are negatively correlated with the growth ratios during the first 0-72 h (or stage 4-7), whereas at later stages, the correlations become positive (Fig. 4A). The change of correlations from negative to positive was further confirmed by calculating, at each time step, the Spearman correlation coefficient (SCC) between the initial sizes 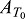 and the growth ratios (*A*_*t*+Δ*t*_/*A*_*t*_)/Δ*t* from all clones. The SCCs indicate that the growth ratios at each time step are negatively correlated at the earlier stages, but become positively correlated at the later stages (Fig. 4B). The transition took place at around stage 6-7, with an exception that the SCC for the sepal wt-a1 has a time delay of ∼36 h. These results also show that there is a temporal variation changing from negative correlation during the size uniformization to positive correlation during the variability promotion stages, as shown in Fig. 4B, meaning that the transition takes place gradually.

### The shift from size uniformization to variability enhancement occurs 24-60 h after a clone initiates from a single cell

A remaining question is whether the switch from size uniformization to variability enhancement relates to the developmental timing of the entire sepal, or relates to the timing at which a clone initiates, as our analysis in the previous sections started from single cells at the first time point of the live imaging series. To answer this question, we considered clones starting from single cells at additional time points during the live imaging sequence of a given sepal, denoted as the starting time *T*_*S*_, at which the set of all individual cells is extracted with their cell walls and areas identified (Fig. 4C, leftmost outlines). Each individual cell defined at *T*_*S*_ therefore serves as the progenitor cell of a particular clone for *t*>*T*_*S*_, i.e. a different choice of *T*_*S*_ (=0 h, 12 h, 24 h, …) results in different choice of clones (Fig. 4C). The different choice of *T*_*S*_ is equivalent to different starting times (denoted by *t*=0 h in Table 1) of the live imaging, which can be arbitrary. We then asked if the signature of size uniformization, i.e. the negative correlations observed in Fig. 4A,B, depends on the choice of *T*_*S*_.

As performed previously (Fig. 4A), we examined how the size of the clone 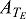 at *t*=*T*_*E*_ (with *T*_*E*_>*T*_*S*_) correlates with the growth ratio 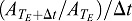 (with Δ*t*=12 h) at each time step (e.g. the dashed line boxes in Fig. 4C for different choices of *T*_*E*_). Fig. 4D provides several examples of the plot 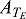 versus 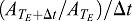, with different choices of *T*_*S*_ and *T*_*E*_. We see that whenever a new clone is initiated from a single cell, it undergoes size uniformization, i.e. there is a negative correlation between clone size and growth rate, indicating that smaller clones grow faster than larger clones (Fig. 4D). Simultaneously, a clone initiated at an earlier time, *T*_*S*_, might have already transitioned to variability enhancement. For example, the clone initiated at 108 h is undergoing size uniformization, whereas the clone initiated at 0 h is undergoing variability enhancement (rightmost panel in Fig. 4D). Because the single cells initiating clones at 108 h are included within the 0 h clones, this constitutes an intricate multiscale system.

We verified that this transition relates to the starting time of the clone with SCCs for different *T*_*S*_ and *T*_*E*_, in which blue and red regions correspond to the size uniformization (A1) and variability enhancement (A3) cases, respectively (Fig. 4E; Fig. S7). Interestingly, the growth transition between A1 and A3 presents as *T*_*S*_ varies. Furthermore, the transition time from the blue to red regions in Fig. 4E is the period in which the corresponding SCCs show that the growth transition from uniformization to variability enhancement gradually shifts to later time depending on the starting time *T_S_*. On average, the transition occurs at ∼60 h for wild type a (wt-a2) [∼24 h for wild type b (wt-b1, wt-b2 and wt-b3) in Fig. S7] after the initiation of the clone from a single cell at *T*_*S*_. This means that the growth transition observed in Fig. 4A and B depends on the age of the clone, i.e. the clones have to grow up to a certain size (or scale) for variability enhancement to happen. Incidentally, this shows that the transition does not depend on the overall developmental timing of the sepal.

### The growth pattern of clones is independent of cell division and stomata differentiation

One of the other possibilities is that size uniformization could depend on the topology of clones, e.g. on cell divisions and the presence of newly built walls. Furthermore, the tipping point *T*_1_ might also relate to the time when epidermal cells acquire a different fate, notably with the differentiation of stomata within clones. To address this issue, we analyzed the correlation between the initial size 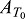 and the growth ratio at each time step (*A*_*t*+Δ*t*_/*A*_*t*_)/Δ*t*, while keeping track of cell divisions in the clones as shown by the colored dots in Fig. 4A. We defined the clones as dividing at time *t* if cell division occurs within the clone during the time step (*t*, *t*+Δ*t*). The dividing clones can also include cells that differentiate and divide into stomata. No significant difference between dividing and nondividing clones from the sepals can be observed (Fig. 4A; Fig. S8). Therefore, we concluded that the growth pattern of clones is independent of cell divisions and differentiation.

### Individual cell growth heterogeneity is positively correlated with the growth of clones at each time step

**Fig. 5.**
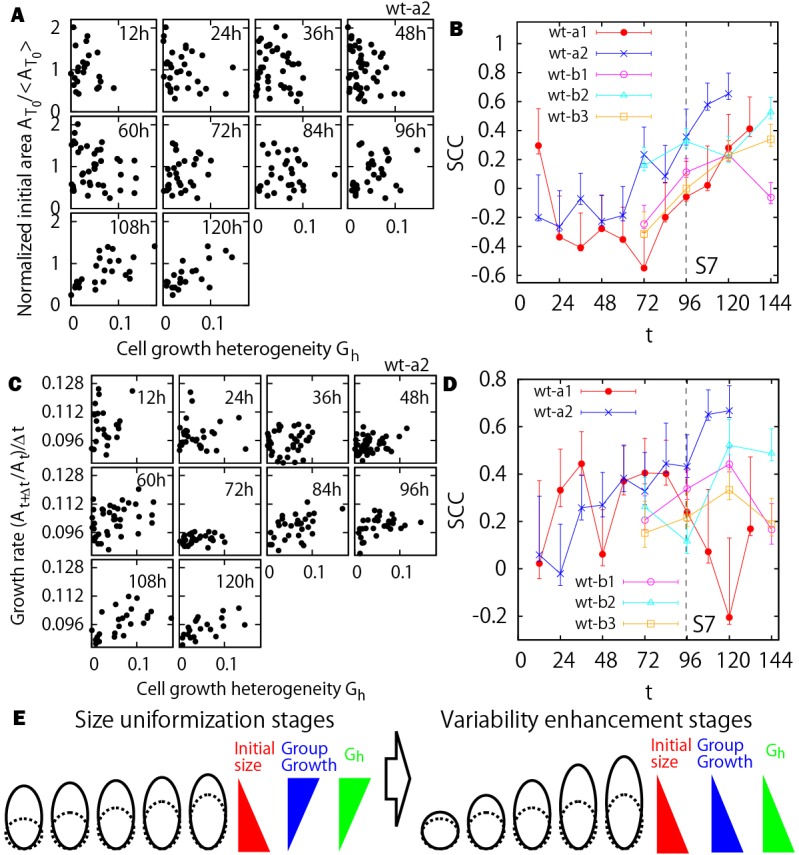
**Increase in cell growth heterogeneity and correlation with initial size of the clone.** (A) Cell growth heterogeneity versus normalized initial area of clones. (B) SCCs of A. (C) Cell growth heterogeneity versus growth ratio of the clone at each time step. (D) SCCs of C. Note that the sepal wt-a1 has less correlation at the later stages, which could relate to the time delay of the growth transition. (E) Schematic illustration of the results for earlier clone size (dashed lines) and later clone size (solid lines).

Because the cause of the tipping point cannot be simply related to a change in cell identity, we next investigated whether growth heterogeneity within the clone might be associated with the shift from uniformization to variability enhancement. To address this question, we quantified the degree of cell growth heterogeneity *G*_*h*_ at time *t*, which is defined as the CV of the cell growth ratio 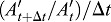 of all individual cells within a specific clone (see Materials and Methods). To determine the role of cell growth heterogeneity in size uniformization, we first asked whether *G*_*h*_ correlates with the initial size 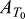 (Fig. 5A,B). We found that the correlation between growth heterogeneity and initial clone size is mostly negative at early stages and becomes positive at the later stages. In other words, a higher level of cell growth heterogeneity exists among the daughter cells for the initially smaller clones at the size uniformization stages, while at the variability enhancement stages the initially larger clones show higher growth heterogeneity (Fig. 5E).

Based on the temporal change of correlation between initial size and *G*_*h*_ (Fig. 5B), and a similar one between the initial size and the growth ratio at each time step (Fig. 4B), a positive correlation between *G*_*h*_ and the growth ratios of the clones at each time step is expected for both the size uniformization and variability enhancement stages. Such positive correlations were confirmed in Fig. 5C and D; namely, a faster growing clone during a time step of Δ*t* (i.e. 12 h in our case) has a larger growth heterogeneity (or variability) among individual cells belonging to the clone. Results from the other sepals are qualitatively similar (Fig. S9). These findings suggest that growth variability at the cellular level could promote the growth of the clone. Alternatively, the fast-growing clones could exhibit more heterogeneity in individual cell growth rates because they have to accommodate the slower growth of neighboring clones. This factor could play an important role in plant cells, because cells are glued to their neighbors and cannot slide against each other as animal cells do. Finally, fast-growing clones are more likely to initiate stomata, which initially grow faster than their immediate neighbors, and can therefore contribute to the increased growth heterogeneity within the clone.

Altogether, these analyses demonstrate that a shift from size uniformization to variability enhancement occurs in growing sepals, which is correlated with a change in growth heterogeneity, rather than cell position or cell identity (Fig. 5E).

## DISCUSSION

To summarize, our results reveal the existence of a growth phase transition between two growth modes in the developing sepal. The size uniformization mode occurs after the initiation of clones, during which initially smaller clones grow faster relative to the larger ones, resulting in a decrease in the area variability of the clones. The variability enhancement mode occurs at the later stages of the clones, during which initially larger clones grow even faster, resulting in an increase in area variability. Although we exclude a role of cell position or cell identity in the shift from size uniformization to variability enhancement, we find that the size uniformization can be, in principle, observed in any clone soon after it initiates from a single cell. One of the surprising observations was that heterogeneity in growth between the cells within the clone correlates with the faster growth of the clone.

The dependence of the size uniformization on the starting time of clone can be interesting in terms of scale dependence, because the size uniformization is observed to occur at smaller cellular scale until the clones reach a larger size, i.e. supracellular scale, in 24-60 h, during which a different growth mechanism of size variability enhancement starts to emerge. This means that the growth behavior at the smaller scale can be different from those at larger scale. To fully understand this multiscale phenomenon, one could investigate the growth with gradual change in spatial scale and to extract the statistical law of growth ratio as a function of scale. This type of growth analysis should be a key prospect to understand growth at the supracellular level.

The conventional meaning of compensation in plant developmental biology is that a balance between cell division and cell growth leads to consistent organ size in mutants or transgenic lines, as a result of changes in cellular size to compensate for changes in the number of cells in the organ (Marshall et al., 2012; Tsukaya, 2003). Here, we detect a similar type of compensation, i.e. size uniformization in the wild type at the clone scale. Recently, size uniformization was observed within sister cells in a single cell cycle in shoot apical meristem (Willis et al., 2016). We note that, in our case, the size uniformization was observed beyond a specific cell cycle, with a much longer time range that spans several flower stages.

Our findings of the long-range temporal correlations (Fig. 2) suggest that a clone still keeps a memory of its initial size, even after a long time. An attractive (but still speculative) hypothesis is that the clone contour (i.e. the initial walls, including the bottom and top walls) provides long-term cues to growth because they are inherited. Thus, it seems that a clone would behave like an individual big cell and act as a small organism that remembers its initial size.

The observed correlation between clone growth and growth heterogeneity implies that the shift rather relies on perception of growth-related parameters. This raises the question of the exact nature of that cue. Growth heterogeneity generates mechanical conflicts (Rebocho et al., 2017) and plant cells are able to respond to such mechanical signals (Hervieux et al., 2016; Uyttewaal et al., 2012). Therefore, our analysis might be consistent with a scenario in which a competence to respond to mechanical cues is regulated in time, making uniformization possible during sepal development.

## MATERIALS AND METHODS

### Plant material and imaging by confocal microscope

The five wild-type sepals were sampled from different laboratories (wt-a1, wt-a2: two sepals imaged every 12 h at ENS Lyon, France; wt-b1, wt-b2, wt-b3: three sepals imaged every 24 h at Max Planck Cologne, Germany). We reanalyzed data already used in Hervieux et al. (2016), in which the average sepal growth pattern was quantified.

In the a1 and a2 series, we used the *pUBQ10::myrYFP* line kindly provided by Raymond Wightman (Sainsbury Laboratory, Cambridge University, UK). In this line, myrYFP corresponds to a YFP that is N-terminally modified with a short peptide that is myristoylated and probably acylated (Yang et al., 2016). Plants were grown under long-day conditions. Staging was determined as indicated in Smyth et al. (1990). Main inflorescence stems (1-2 cm long) were cut from the plant. To access young buds, the first 10-15 flowers were dissected. The young buds were imaged with an SP8 laser-scanning confocal microscope (Leica) using long-distance 40× (NA 0.8) water-dipping objectives. During time-lapse imaging, plants were kept in one-half Murashige and Skoog medium with plant protective medium (1 ml/l), and imaged every 24 h for up to 8 days.

In the b1, b2 and b3 series, early stages of floral development (from 3 to 6) were determined based on comparison of floral bud morphology with the stages proposed by Smyth et al. (1990). The timing of development from stage 3 to 6 corresponded to the one published for similar growth conditions (Smyth et al., 1990). Therefore, later developmental stages of the same flowers were determined by comparing the timing from stage 6 (bud closure) with the timing of development reported by Smyth and coworkers.

### Extraction of cell surface and cell growth extraction using MGX software

To investigate cell growth, we used an open source application, MGX, for the visualization and analysis of a fluorescence data (Barbier de Reuille et al., 2015). As described by Willis et al. (2016), a key strength of MGX is the ability to summarize 3D fluorescence data as a curved surface image. From the fluorescence data, MGX allows us to detect the outermost surface indicating the 3D organ shape. After creating the surface mesh, which is the aggregation of small triangles along with the organ surface, we can also specify the position of cell walls (cell outlines in the main text) using a membrane fluorescence which has a strong brightness at the cell walls. Then, we can calculate a specific cell surface area to sum up the total area of small triangles within a cell. We also have lineage information combining a data set observed at different times. In this study, we first detected a cell outline at the first time frame and kept track of the outline in which cells have the same lineage as the initial cell. The growth in a clone can be calculated as the total cell area of a clone at time *t*+Δ*t* over the total cell area of a clone at time *t*, i.e. *A*_*t*+Δ*t*_/*A*_*t*_.

### Bootstrap method to estimate the first and third quartiles of the CV

We calculated the first and third quartiles of the CV using the bootstrap method. The bootstrap method can be summarized as ‘random draw with replacement’, which allows us to measure the accuracy of the statistics. For the sake of a general description, suppose that we have an observed valuable X (the sample number is N) and its CV (standard deviation of X/mean of X). We first create a ‘bootstrapped data set X^B^’. That is, we randomly draw samples N times from data set X with replacement and set it as X^B-1^ for the first trial. We continue in the same way and create the data sets (X^B-1^, X^B-2^, …, X^B-1000^). Using these data sets, we can obtain a bunch of ‘bootstrapped CV’ (CV^B-1^, CV^B-2^, …, CV^B-1000^). We then calculate the first and third quartiles of the distribution of the bootstrapped CV.

### A phenomenological model to verify the linear relationship between early clone size and later clone size

Using the growth rate information, we construct a simple phenomenological model as follows. Based on the previous work (Tauriello et al., 2015), we fit the growth curve of the area *A*_*t*_ with the sigmoidal functional form, i.e. *f*(*t*)=(*K*/(1+exp *r*(*t*_*c*_−*t*)))+*C* (Fig. S2A). In this setup, the growth rate *f*^′^(*t*) is the combination of the linear and the exponential function. From the fitting parameters (Fig. S2B), we obtain a model as a function of the initial size, *A*_0_, as 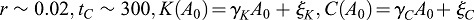, where *γ*_*K*_=264, *γ*_*C*_=2.04 from the fitting and the random variable *ξ*_*K*_, *ξ*_*C*_ are uniformly sampled from the range (–10^4^, 10^4^) and (–10^2^, 10^2^), respectively, being consistent with the observed range of noise. The phenomenological model shows the linear relationship between 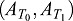 and 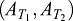, respectively (Fig. S2C), being qualitatively the same as in the previous linear regression analysis (Fig. 2D,E). That means that the linear relationship is consistent with the sigmoid functional form with the linear dependence of the parameter K (total growth amount of clones) and the parameter *C* (area of clone at the very beginning). Furthermore, we emphasize that the sigmoidal growth curve alone does not give rise to linear relationship in Fig. 2D and E. The linear relationship is a combined effect from both the sigmoidal growth and stochasticity in the growth curves, i.e. *K*(*A*_0_) and *C*(*A*_0_), discussed above, among different clones.

### Quantification of cell growth heterogeneity

The cell growth heterogeneity *G*_*h*_ at time *t* is calculated by the CV of the individual cell growth 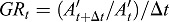 within a specific clone, where 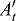 is the surface area of the individual cell at time *t*. Suppose that we have M individual cell growth rates, *GR*_*t*_(*C*_1_), at time *t* within a specific clone *C*_1_, that we have the CV, *CV*_1_(*C*_1_), for the specific clone data set 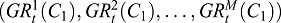. Likewise, if we applied this process to *K* clones, we obtain the data set of the CV (*CV*_1_(*C*_1_), *CV*_2_(*C*_2_), …, *CV*_*K*_(*C*_*K*_)). Then, the term ‘cell growth heterogeneity’ in the main text is defined as the averaged value of the data set (*CV*_1_(*C*_1_), *CV*_2_(*C*_2_), …, *CV*_*K*_(*C*_*K*_)).

## Supplementary Material

Supplementary information

Supplementary information
