# Supplementary material for: Clones of cells switch from reduction to enhancement of size variability in Arabidopsis sepals
Source: Development. 2017 Dec 1;144(23):4398–405. doi: 10.1242/dev.153999 (PMC5769632; doi:10.1242/dev.153999)
Supplement: Supplementary information [file develop-144-153999-s1.pdf]

Fig. S1A

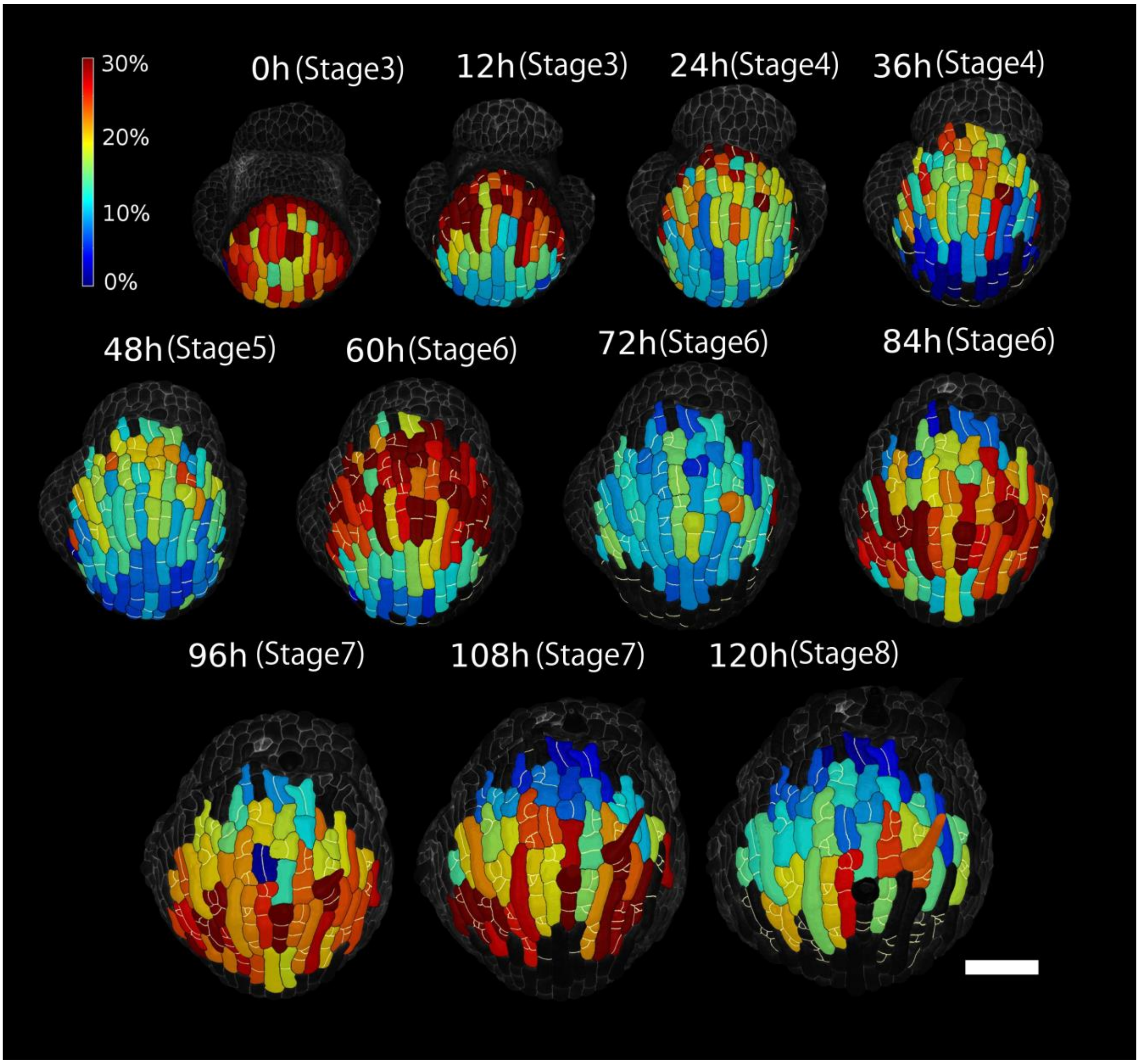
30%
20%
10%
0%
0h (Stage3)
12h (Stage3)
24h (Stage4)
36h (Stage4)
48h (Stage5)
60h (Stage6)
72h (Stage6)
84h (Stage6)
96h (Stage7)
108h (Stage7)
120h (Stage8)

Fig. S1B

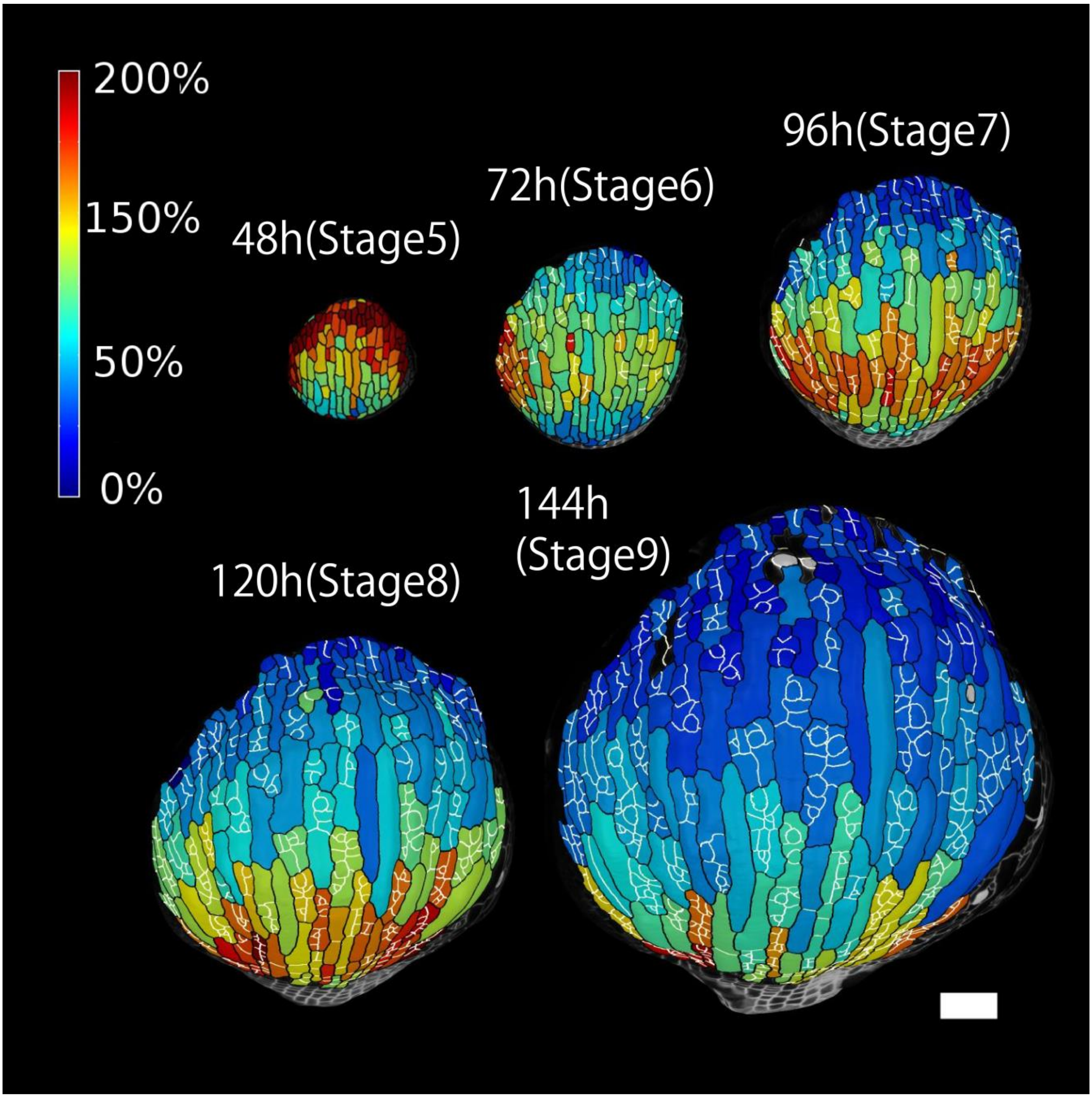

Development • Supplementary information

Fig. S1C

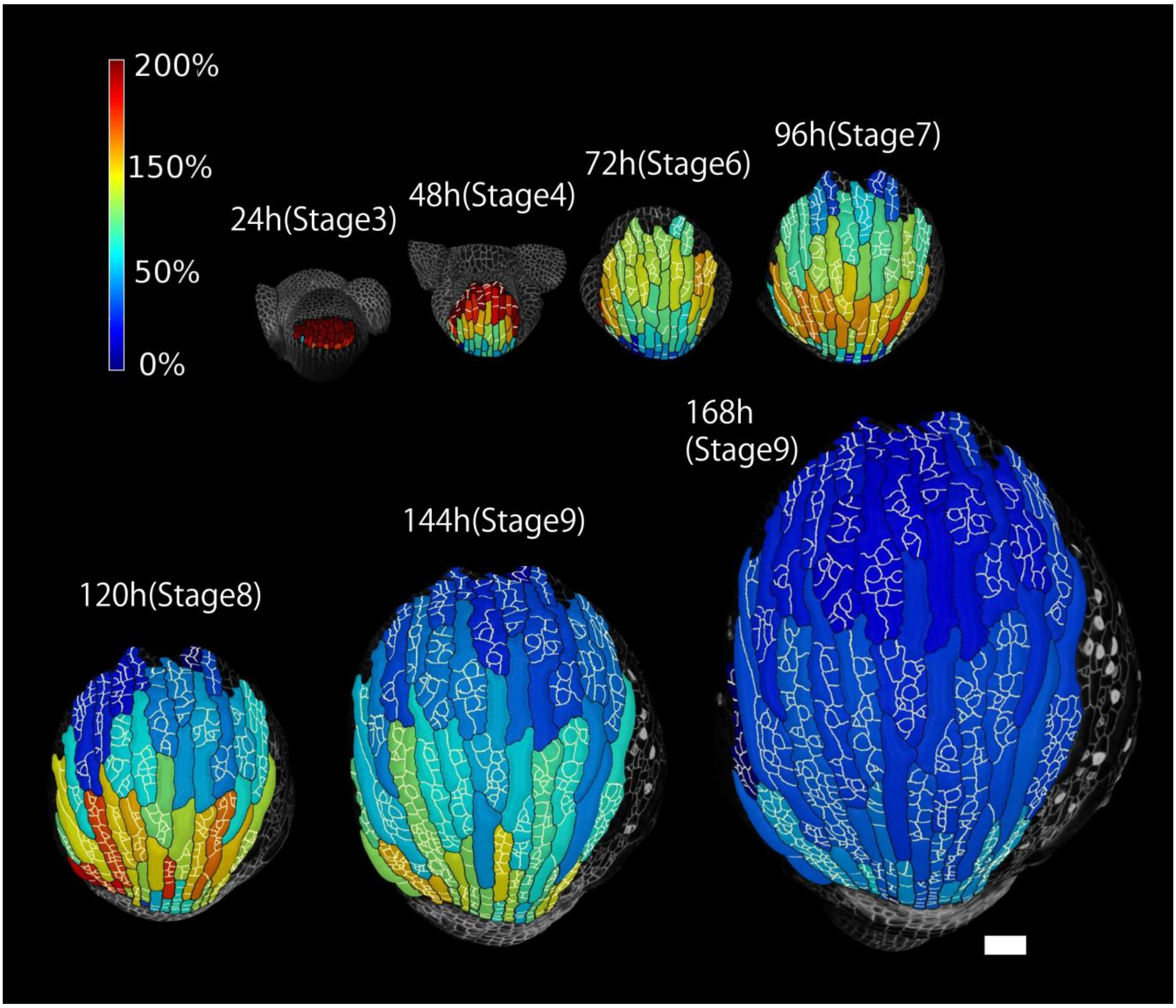
200%
150%
50%
0%
24h(Stage3)
48h(Stage4)
72h(Stage6)
96h(Stage7)
168h
(Stage9)
144h(Stage9)
120h(Stage8)

Fig. S1D

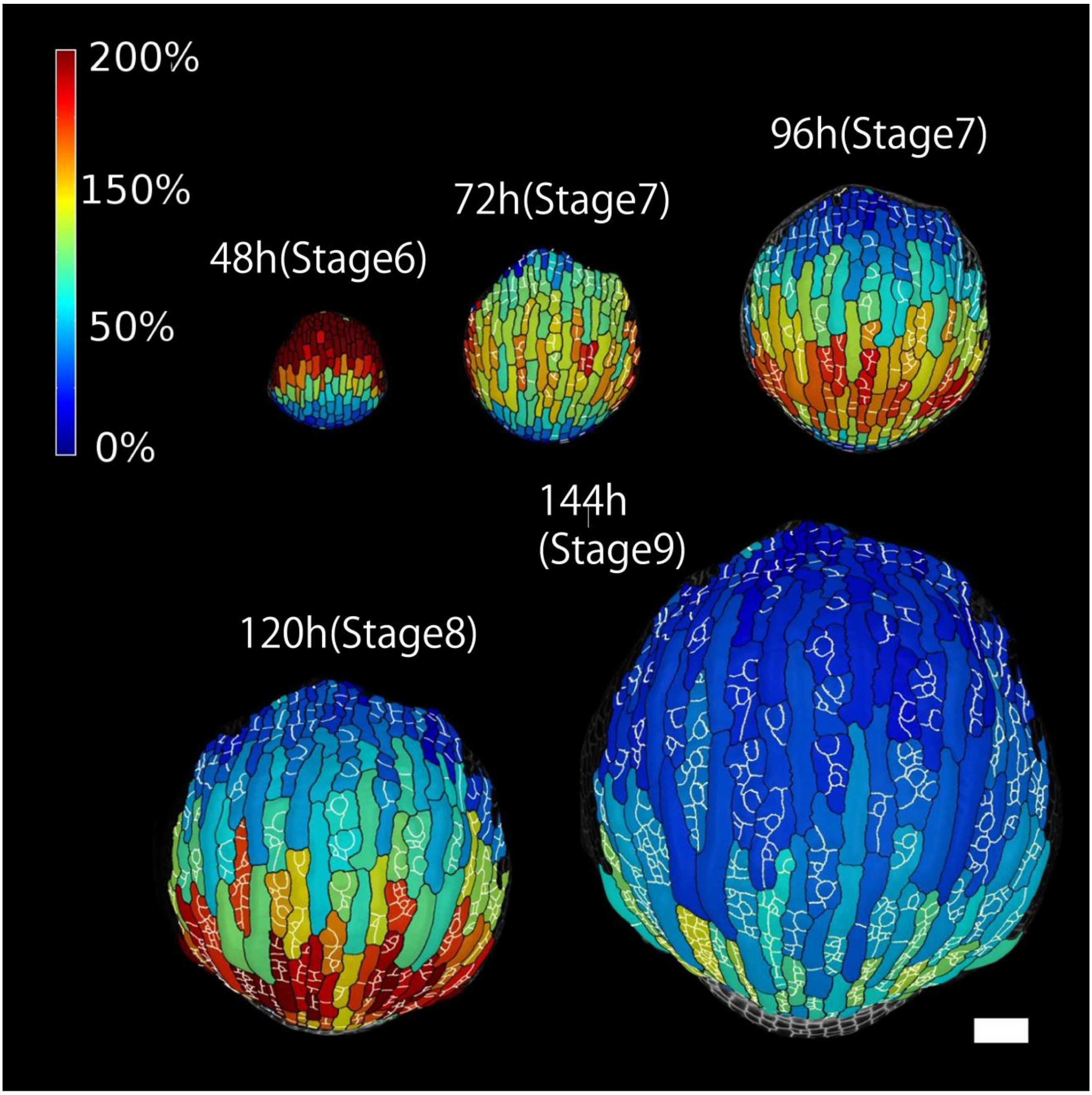

Fig. S1A-D. Heat map of areal growth of clones $(A_{t+\Delta t} - A_t)/A_t \times 100$ (%) over consecutive $12h$ intervals for flower wt-a2 (S1A), and consecutive $24h$ intervals for flower wt-b1, wt-b2 and wt-b3 (S1B-S1D). Scale bars are $50\mu m$. The clones and the new cell walls built after 0h are outlined in black and white, respectively.

Fig. S2.

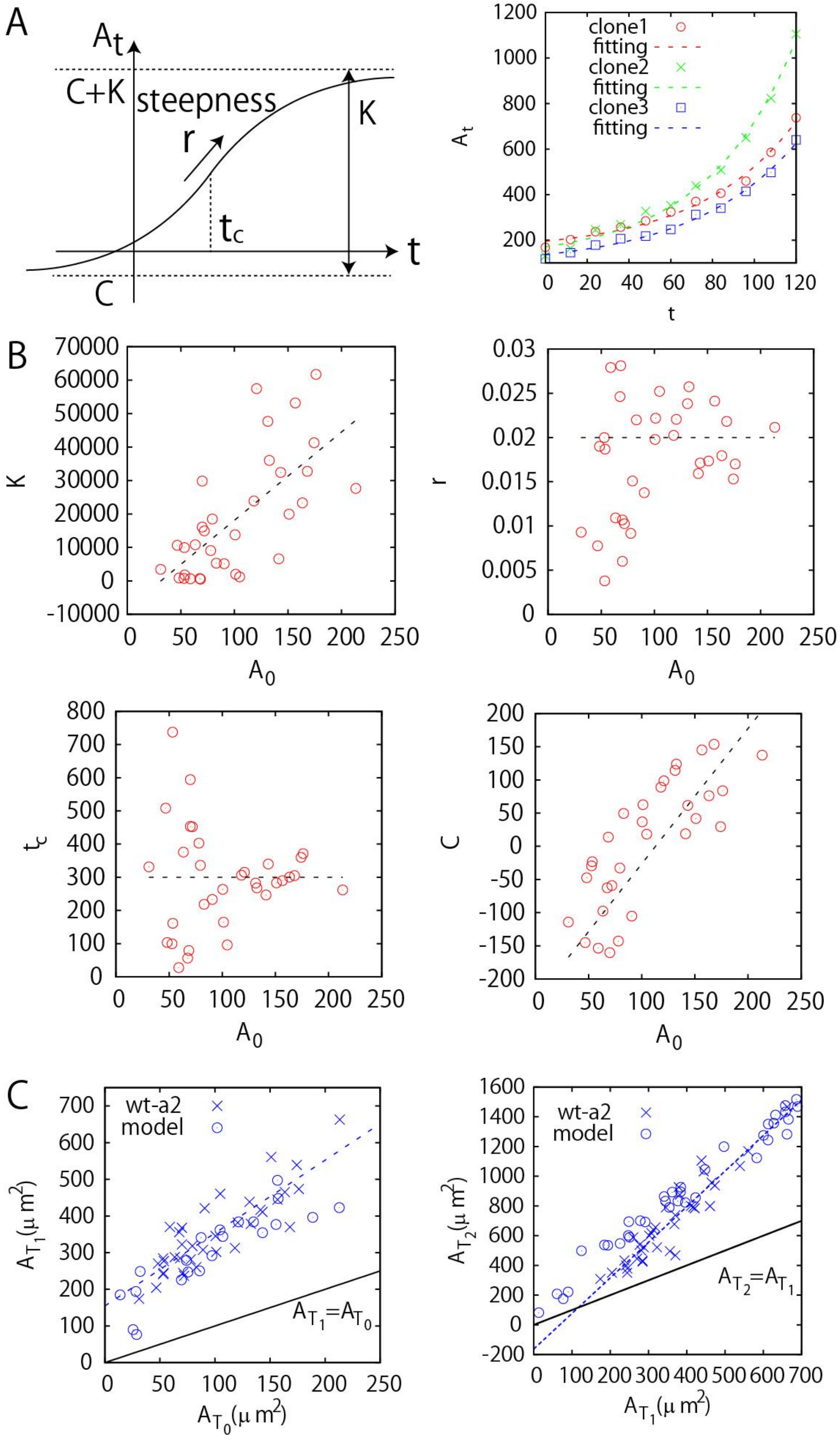
A
At
C+K
steepness
r
K
tc
t
C
clone1
fitting
clone2
fitting
clone3
fitting
At
t
B
K
A0
r
A0
tc
A0
C
A0
C
wt-a2
model
AT1(μ m2)
AT0(μ m2)
AT1=AT0
wt-a2
model
AT2(μ m2)
AT1(μ m2)
AT2=AT1

Fig. S2. (A) (Left panel) Schematic of the sigmoidal functional form $f(t) = \frac{K}{1+\exp r(t_c - t)} + C$ where r is the steepness of the curve around the half time $t = t_c$, K is the total growth amount and C is the clone area in the very beginning. (Right panel) Fitting results of the growth curve of the area A_t. (B) Four fitting parameters with the phenomenological model (dotted line). (C) Comparion between the model and the data from Fig. 2D and 2E.

Development • Supplementary information

Fig. S3.

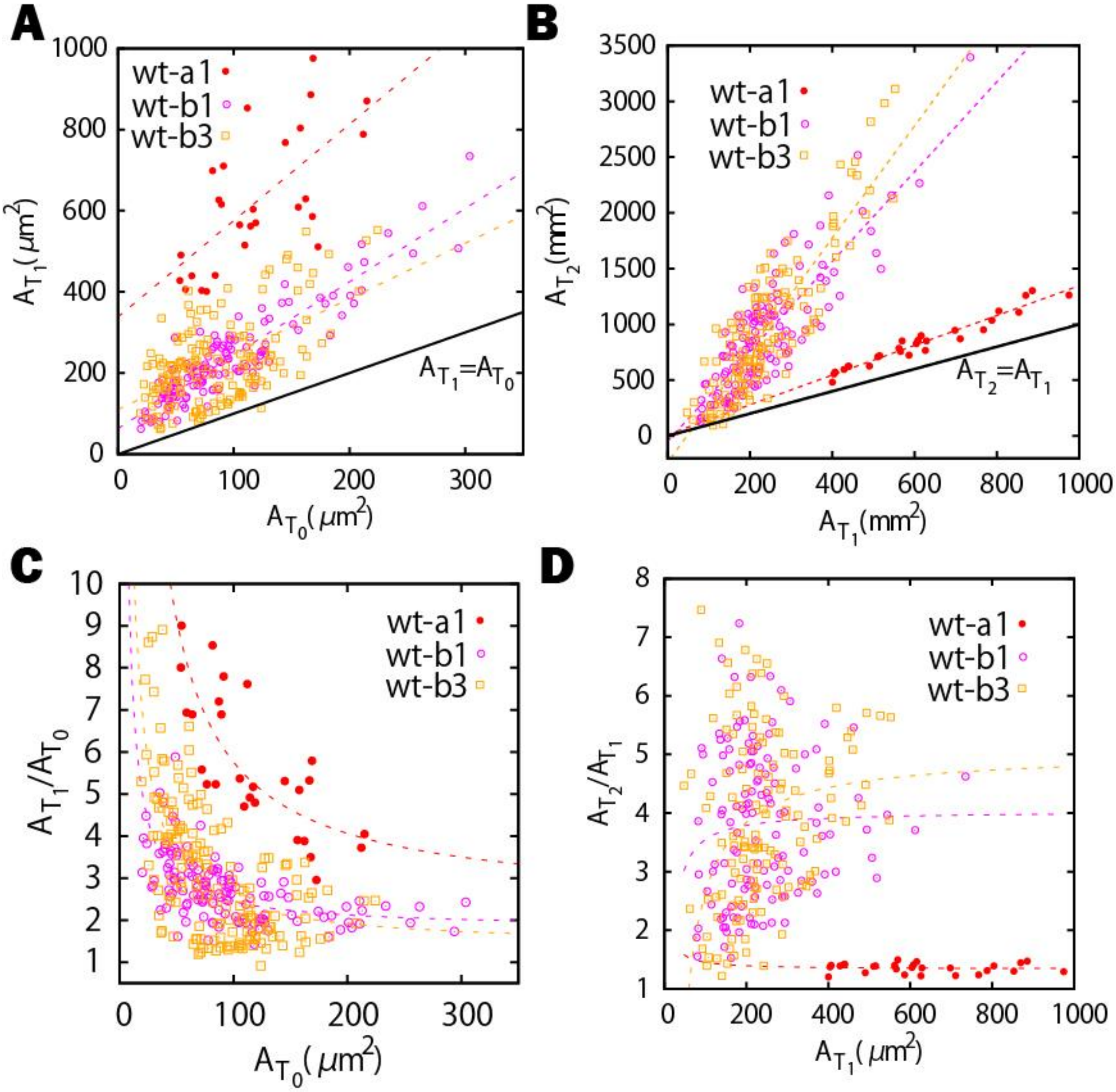

Fig. S3. (A-B) A_{T_E} versus A_{T_L} plot for the clones at T_0 versus those at T_1 (A), and at T_1 versus those at T_2 (B). (C-D) A_{T_E} versus A_{T_L}/A_{T_E} plot for the clones at T_0 versus those at T_1 (C), and at T_1 versus those at T_2 (D). Error bars represent the 50%-confidence interval (see Materials and Methods).

Development • Supplementary information

Fig. S4.

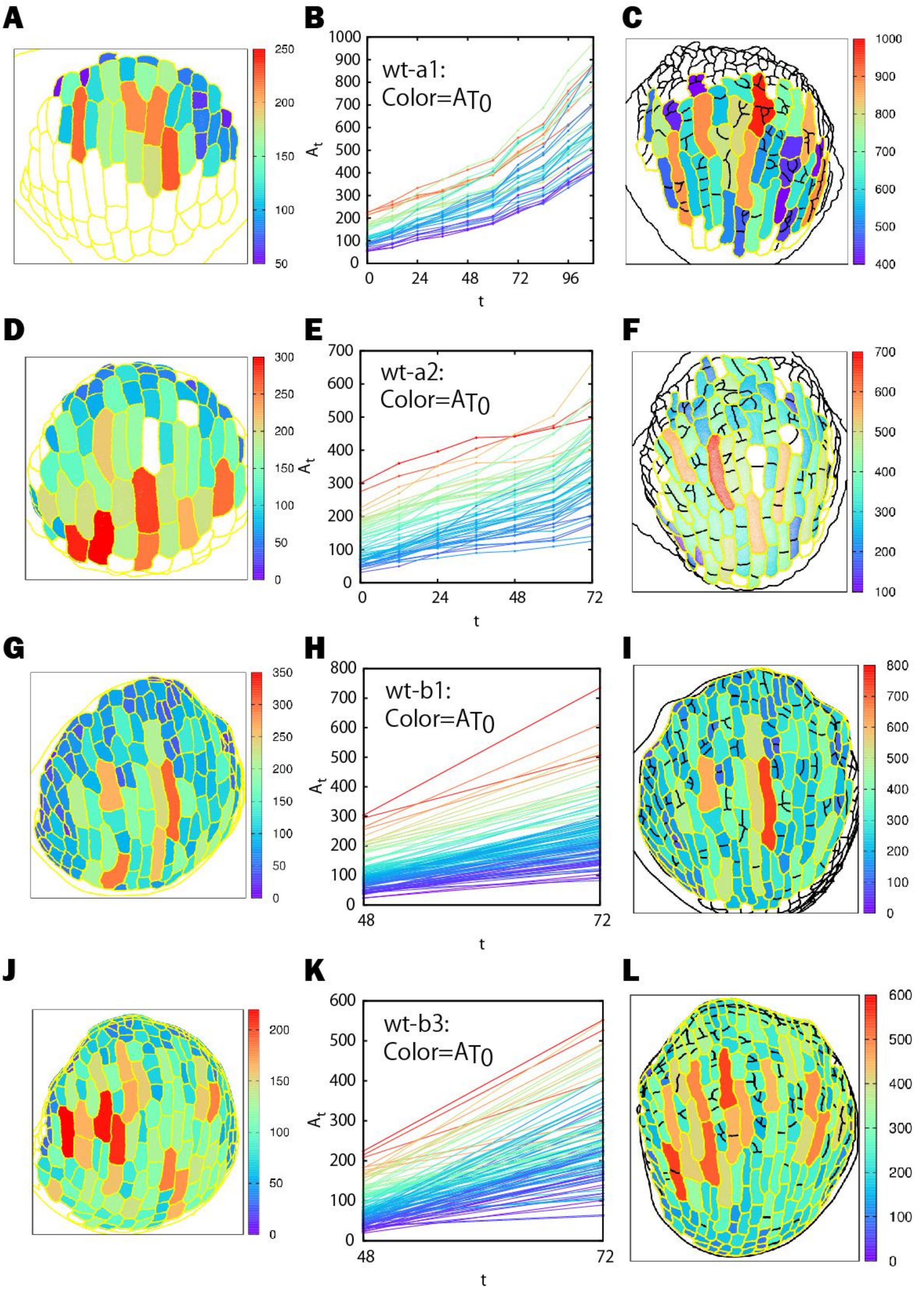

Fig. S4. (A,D,G,J) Heat map of the initial clone area A_{T_0} (μm^2). (B,E,H,K) Growth curves of clones colored according to their initial size A_{T_0}. (C,F,I,L) Heat map of the size of the clones A_{T_1} (μm^2).

Development • Supplementary information

Fig. S5.

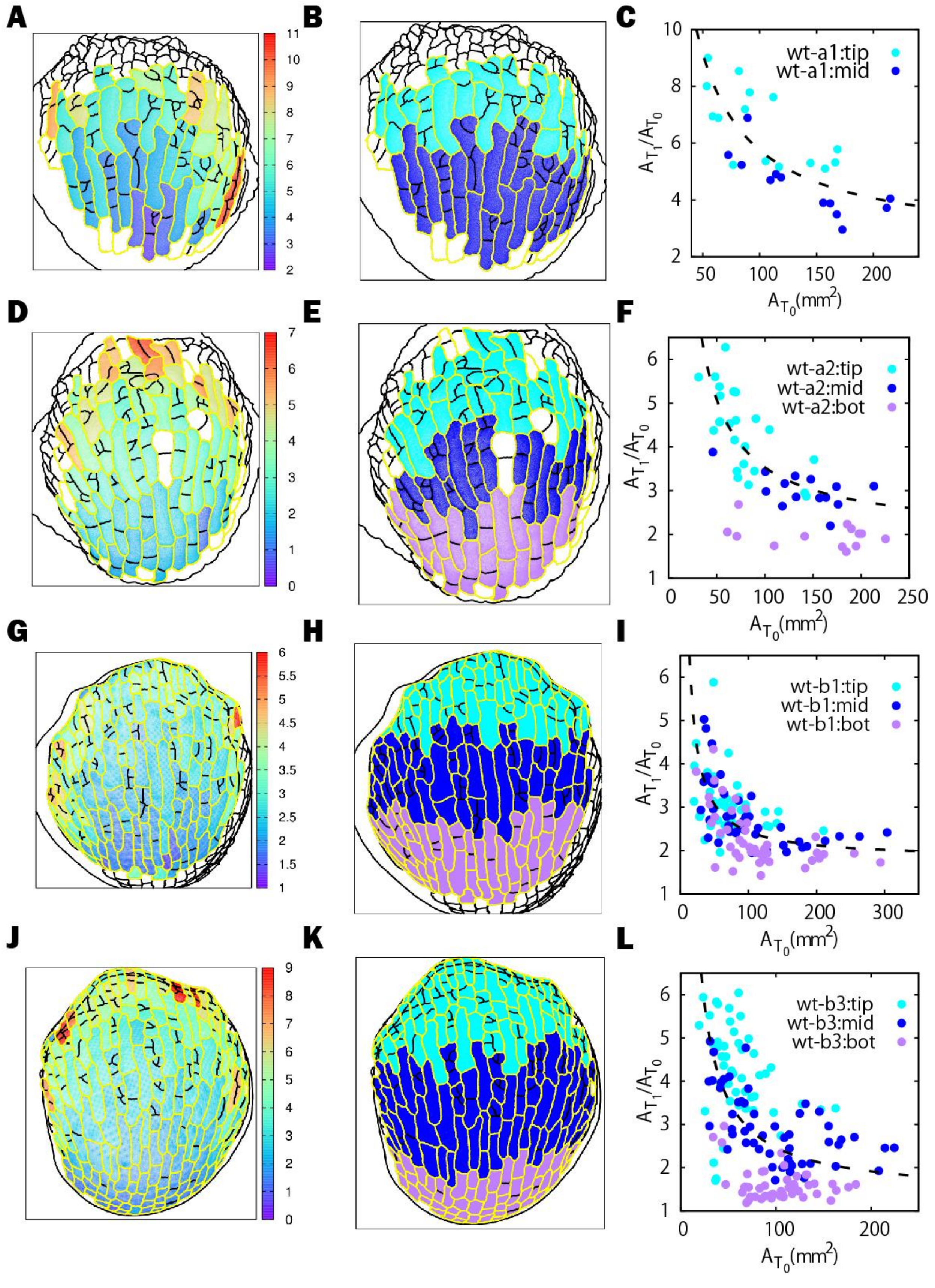

Fig. S5. (A,D,G,J) Heat map of the cumulative growth ratio A_{T_1}/A_{T_0} showing a continuous spatial trend from tip to bottom. (B,E,H,K) Classification of tip (cyan), middle (blue) and bottom (purple) regions. (C,F,I,L) Plot of cumulative growth ratios A_{T_1}/A_{T_0} versus initial area A_{T_0} for clones in each region.

Development • Supplementary information

Fig. S6.

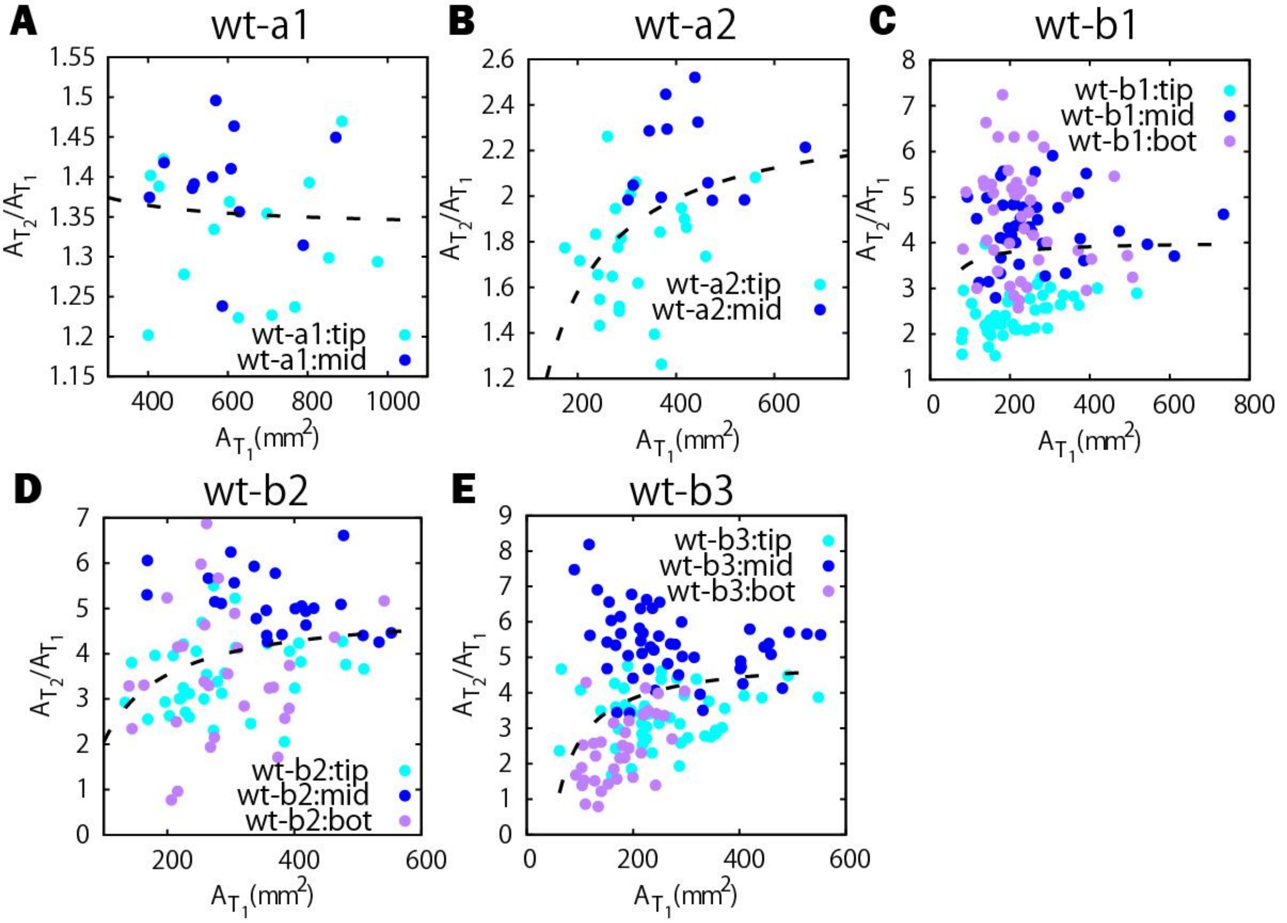

Fig. S6. (A-E) Plot of cumulative growth ratios A_{T_2}/A_{T_1} versus initial area A_{T_1} for clones in each region for wt-a1, wt-a2, wt-b1, wt-b2 and wt-b3.

Development • Supplementary information

Fig. S7.

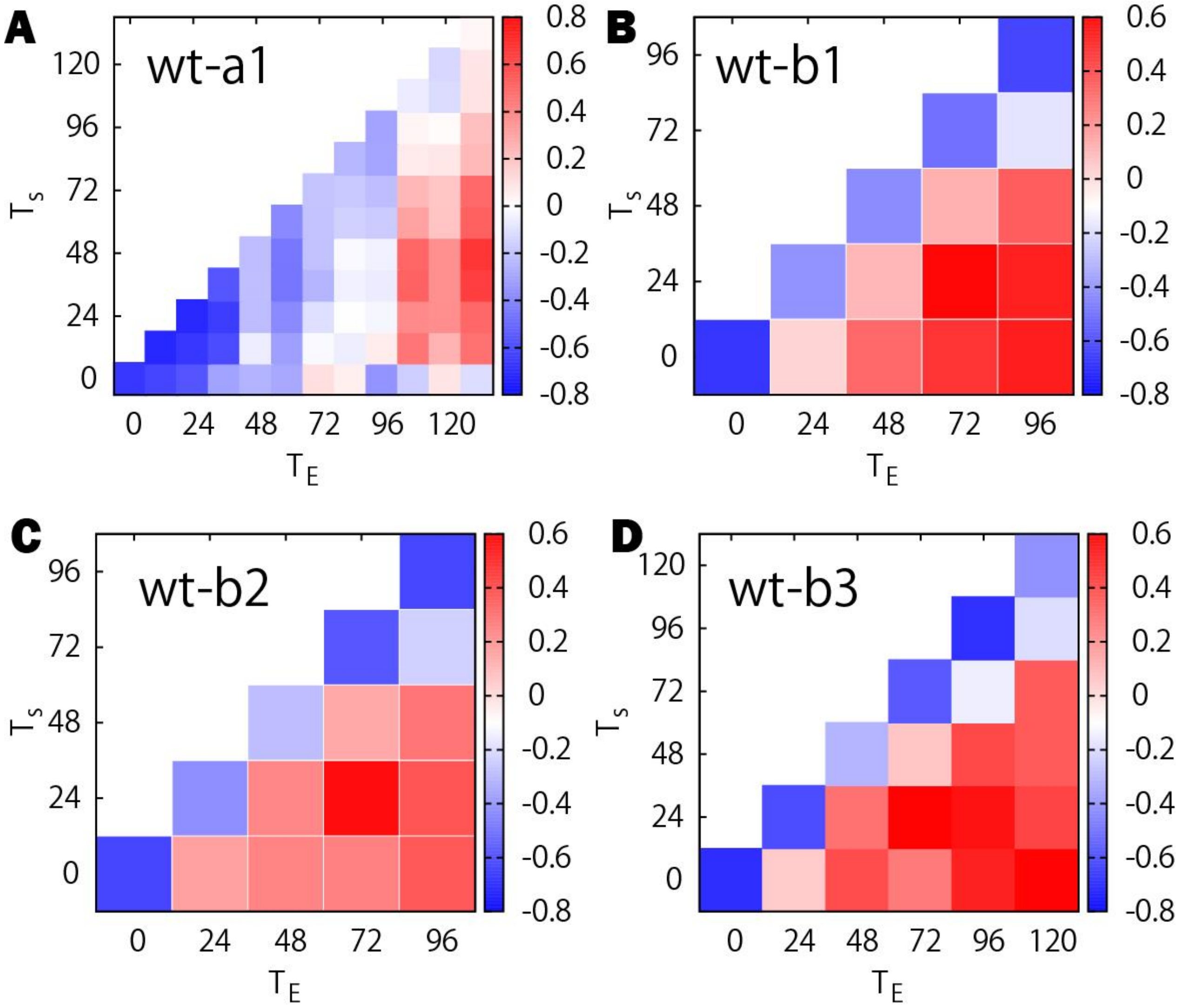

Fig. S7. (A-D) Spearman correlation coefficient between size and growth ratio of the clone for different choice of T_s and T_E for the sepal wt-a1, wt-b1, wt-b2 and wt-b3, respectively.

Fig. S8.

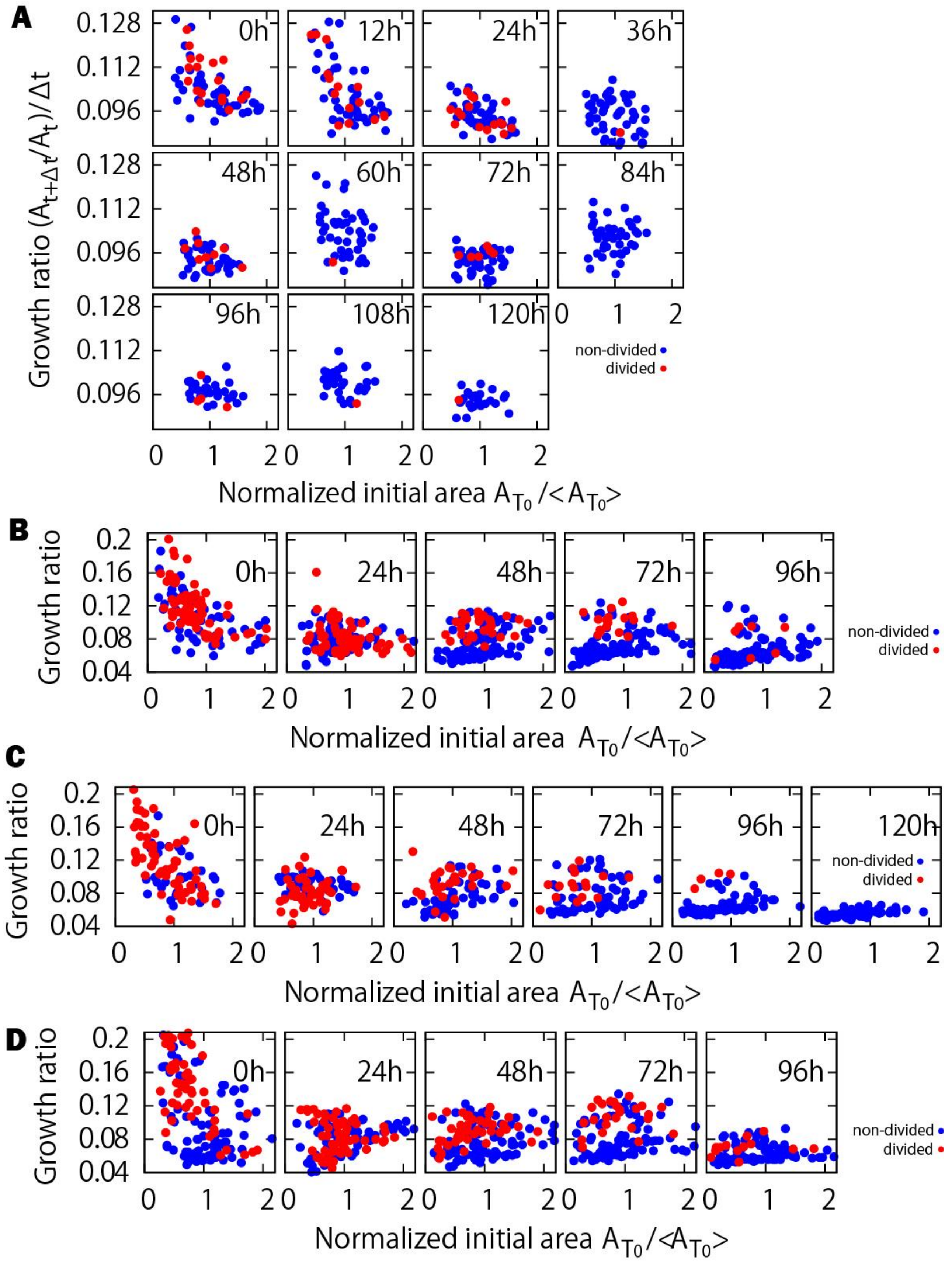

Fig. S8. (A-D) Plot of growth ratio of the clone at each time step $(A_{t+\Delta t}/A_t)/\Delta t$ versus normalized initial clone area $A_{T_0}/\langle A_{T_0}\rangle$ for the sepal wt-a1, wt-b1, wt-b2 and wt-b3, respectively.

Development • Supplementary information

Fig. S9.

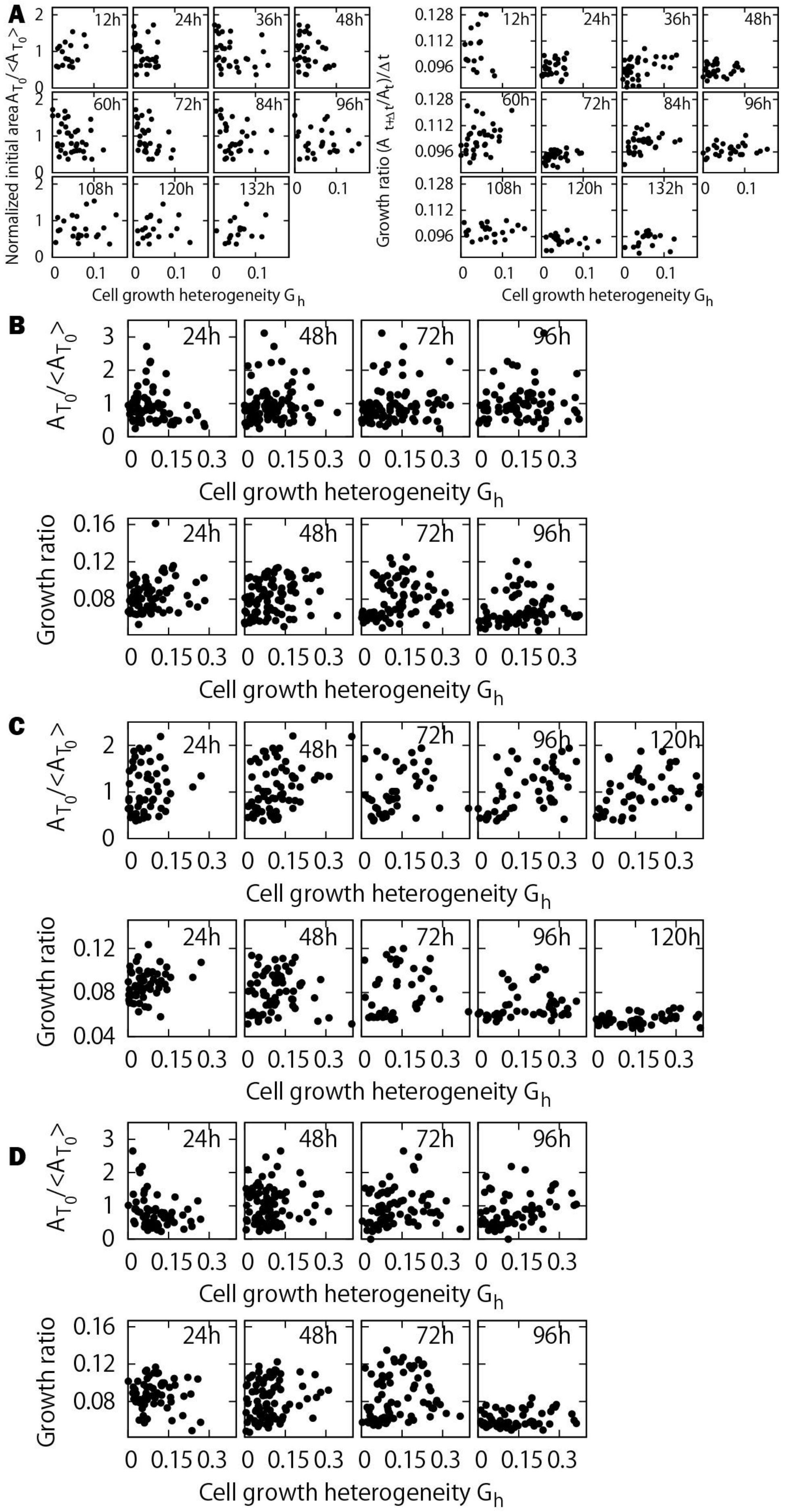

Development • Supplementary information

Fig. S9. (A-D) Cell growth heterogeneity versus normalized initial clone area and cell growth heterogeneity versus growth ratio of the clone at each time step for the sepal wt-a1, wt-b1, wt-b2 and wt-b3, respectively.
